# Locally delivered dexpanthenol enhances bone regeneration in a rabbit defect model: Implications for oral and maxillofacial surgery

**DOI:** 10.1186/s12903-026-08702-y

**Published:** 2026-05-22

**Authors:** Mustafa Isleyen, Muge Cina, Bunyamin Altıntas, Ozlem Ozmen

**Affiliations:** 1 Department of Oral and Maxillofacial Surgery, Faculty of Dentistry, Burdur Mehmet Akif Ersoy University, Burdur, Türkiye; 2 Department of Oral and Maxillofacial Surgery, Faculty of Dentistry, Suleyman Demirel University, Isparta, Türkiye; 3 Department of Pathology, Faculty of Veterinary Medicine, Burdur Mehmet Akif Ersoy University, Burdur, Türkiye

**Keywords:** Antioxidants, Bone defect, Bone regeneration, Dexpanthenol, Gelatin sponge

## Abstract

**Background:**

Bone regeneration plays a critical role in oral and maxillofacial surgical procedures, including implant site preparation, periodontal defect management, and guided bone regeneration. Dexpanthenol (DXP), a well-tolerated derivative of pantothenic acid with antioxidant and wound-healing properties, has demonstrated beneficial effects in soft tissue repair; however, its potential role in bone regeneration remains insufficiently explored. Therefore, this study aimed to evaluate the effects of locally delivered DXP on bone healing using a standardized rabbit defect model.

**Methods:**

This in vivo experimental study employed an intra-animal paired tibial defect model in eight adult male New Zealand White rabbits. Two standardized monocortical defects (6 mm in diameter and 8 mm in depth) were created in the right tibia of each animal. One defect was treated with a gelatin sponge (GS) (control), while the adjacent defect received DXP-impregnated GS. After a 30-day healing period, histomorphometric analysis and immunohistochemical evaluation of vascular endothelial growth factor (VEGF) expression were performed.

**Results:**

DXP-treated defects exhibited significantly improved bone healing parameters compared to controls, including increased total healing area, higher defect closure rate, new bone formation, elevated osteoblast counts, and enhanced collagen deposition (*P* < 0.001 for all). VEGF expression was also significantly higher in the DXP group (*P* < 0.001), suggesting increased angiogenesis-related activity. No significant difference was observed in osteoclast counts (*P* > 0.05).

**Conclusions:**

Within the limitations of this experimental model, locally delivered dexpanthenol was associated with enhanced bone formation healing and increased VEGF expression. However, given the use of a non-craniofacial defect model and the absence of mechanistic analyses, these findings should be interpreted with caution. Further studies using clinically relevant models and molecular approaches are required to clarify the underlying mechanisms and translational potential of DXP in bone regeneration.

## Introduction

Bone healing is a highly coordinated biological process essential for restoring skeletal integrity following trauma, pathological conditions, or surgical interventions [1]. In oral and maxillofacial surgery, achieving predictable and rapid bone regeneration remains a persistent clinical challenge, particularly in the management of cystic defects, extraction sockets of impacted teeth, and dental implant site preparation. Successful bone repair relies on a tightly regulated interplay between cellular proliferation, angiogenesis, extracellular matrix deposition, and subsequent remodeling, all of which are modulated by local microenvironmental factors and systemic signaling pathways [2, 3].

Despite advances in regenerative strategies, the management of localized bone defects remains suboptimal. Current approaches, including growth factor delivery, stem cell–based therapies, and bioactive scaffolds, aim to enhance osteoblast differentiation, matrix synthesis, and vascularization; however, their clinical translation remains constrained by high costs, complex preparation protocols, and variable outcomes [4]. Therefore, there is a compelling need for alternative, cost-effective agents that can safely and effectively promote bone regeneration.

Dexpanthenol (DXP), a stable alcohol analog of pantothenic acid (vitamin B5), plays a critical role in cellular metabolism through its conversion to coenzyme A, thereby influencing cellular proliferation and tissue repair processes [5]. It has been widely recognized for its potent wound-healing properties, particularly in cutaneous and mucosal tissues, where it exerts anti-inflammatory effects, enhances fibroblast proliferation, stimulates collagen synthesis, and promotes angiogenesis [6, 7]. These biological activities suggest that DXP may favorably modulate key pathways involved in bone regeneration.

Emerging evidence indicates that DXP-based formulations, including nanofiber delivery systems, can accelerate wound closure, enhance re-epithelialization, and promote collagen deposition in experimental models, highlighting their role in regulating tissue regeneration and cellular proliferation [8]. Consistent with these findings, DXP has been shown to improve healing outcomes in various soft tissues, primarily through modulation of inflammatory responses and stimulation of cellular proliferation [9, 10].

Despite these promising attributes, the direct effects of DXP on bone tissue particularly in the context of osteogenesis and defect healing remain insufficiently characterized. This represents a significant gap in the current literature, as the translation of DXP’s well-established regenerative potential from soft tissues to bone has not been adequately investigated.

Numerous biomaterial-based strategies have been explored to enhance bone regeneration, including growth factor delivery systems, stem cell-based therapies, bioactive scaffolds, and drug-loaded matrices. These approaches aim to modulate key processes such as osteoblast differentiation, extracellular matrix production, and angiogenesis, which are critical for successful bone healing. However, their clinical applicability is often limited by complexity, cost, and variable outcomes, underscoring the need for alternative therapeutic agents.

Accordingly, the present study aimed to evaluate the effects of locally administered DXP on bone healing using a standardized rabbit tibial defect model, which provides a relevant translational framework for maxillofacial bone regeneration. We hypothesized that local DXP applications would enhance early-stage bone repair by promoting osteoblastic activity, collagen deposition, and angiogenesis within the defect area. The null hypothesis (H₀) was that DXP has no significant effect on bone healing compared with the control group, whereas the alternative hypothesis (H₁) was that DXP would significantly enhance osteogenesis and vascularization.

## Methods

### Experimental design

This in vivo experimental study employed an intra-animal paired tibial defect model using eight adult male New Zealand White rabbits (2–3 kg) as a translational approach for maxillofacial bone regeneration. Ethical approval was obtained from the Burdur Mehmet Akif Ersoy University Local Ethics Committee for Animal Experiments (decision no. 125/1331, dated 18.07.2024) and all procedures were conducted in accordance with the ARRIVE Guidelines 2.0 [11]. The rabbits were obtained from the Experimental Animal Production and Research Center of the same institution, where all experimental procedures were performed. Animals were housed individually under standardized conditions (24 °C, 12-hour light/dark cycle) with ad libitum access to food and water. Body weight was recorded weekly throughout the study period.

The sample size (*n* = 8) was determined based on previously published comparable rabbit bone defect models [12]. No formal a priori power analysis was conducted; instead, the number of animals was selected in line with similar preclinical studies, while adhering to the principles of reduction and ethical refinement in animal experimentation.

For the creation of bone defects, two standardized monocortical defects were prepared in the right tibia of each rabbit using a trephine burr. This intra-animal paired design allowed each animal to serve as its own control, thereby minimizing inter-individual variability and increasing statistical efficiency. One defect was assigned to the control condition (gelatin sponge [GS] alone), while the adjacent defect received DXP-impregnated GS.

**Fig. 1:**
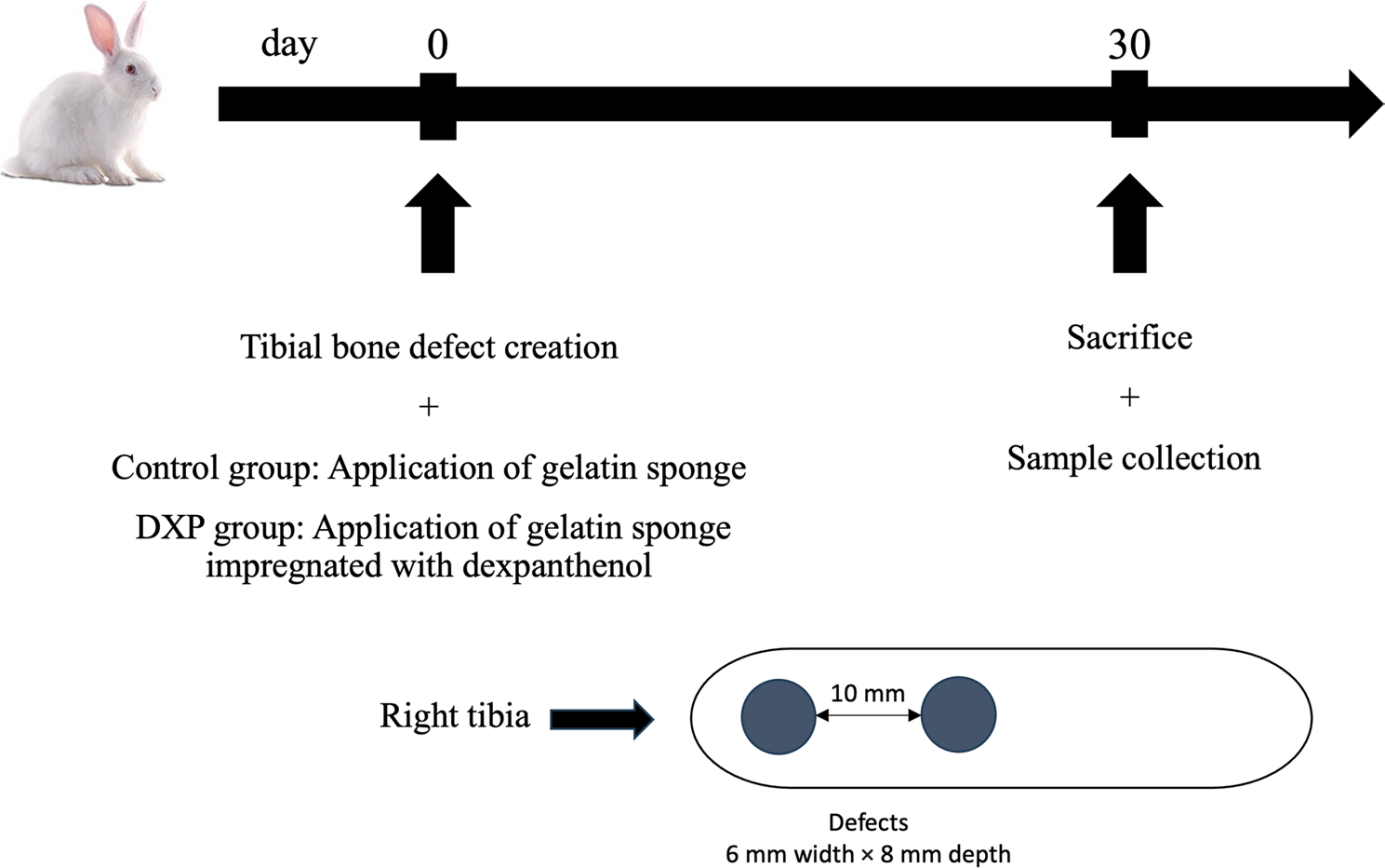
Experimental design and schematic overview. Schematic representation of the experimental design showing the creation of two standardized defects in the right tibia of each rabbit, allocation of treatments (gelatin sponge [GS] vs. dexpanthenol [DXP]-impregnated GS), and the 30-day healing period prior to sample collection

In the control group (*n* = 8 defects), the defects were filled with a GS (lyophilized hydrolyzed collagen, Surgispon). In the experimental group (*n* = 8 defects), the defects were filled with a DXP-impregnated GS (Bepanthene ampoule, 500 mg/2 ml; Roche, Berlin, Germany) (Fig. 1). The tibial defect model was selected due to its reproducibility and translational relevance to oral and maxillofacial regeneration.

It should be noted that the use of gelatin sponge in both groups may exert inherent osteoconductive effects; therefore, the observed outcomes likely reflect the additional modulatory contribution of dexpanthenol rather than an isolated effect.

### Preparation of DXP-impregnated GS

DXP-impregnated GSs were prepared according to a protocol adapted from Zhang et al. [13]. Briefly, preweighed lyophilized GS samples (W_0_) were immersed in DXP solution at room temperature for 10 min to allow complete swelling. After incubation, excess surface solution was gently removed using filter paper, and the swollen scaffolds were reweighed (W_1_).

The swelling ratio was calculated using the formula: $\left(\mathrm{W}_{1}-\mathrm{W}_{0}\right)/\;\mathrm{W}_{0}\times100\%$. The mean initial dry weight of GS was 4 mg, which increased to 23.5 mg after immersion, corresponding to a swelling ratio of 487.5%. Based on the concentration of the DXP solution (250 mg/mL), each scaffold was estimated to absorb approximately 19.5 µL of solution, resulting in an approximate loading of 4.9 mg DXP per defect.

This impregnation process was standardized and repeated for each scaffold to ensure reproducibility and consistency across all experimental samples. To minimize potential confounding effects related to differential fluid uptake, control GS specimens were subjected to an identical soaking procedure using the vehicle solution (sterile physiological saline) without the active compound, ensuring comparable hydration, handling, and mechanical characteristics between groups. Although this approach provided controlled and uniform drug loading, no in vitro or in vivo release kinetics analysis was performed, which should be considered a methodological limitation.

### Surgical method

All surgical procedures were performed under general anesthesia induced by intramuscular administration of ketamine hydrochloride (35 mg/kg; Ketax, Vem Drug, Türkiye) and xylazine hydrochloride (5 mg/kg; Rompun, Bayer, Germany). Adequate depth of anesthesia was confirmed by the absence of the eyelid reflex.The operative site was shaved and disinfected with povidone-iodine solution (Baticonol, Dermosept, Türkiye). To enhance postoperative analgesia, 2% lidocaine was infiltrated locally at the surgical site.

**Fig. 2:**
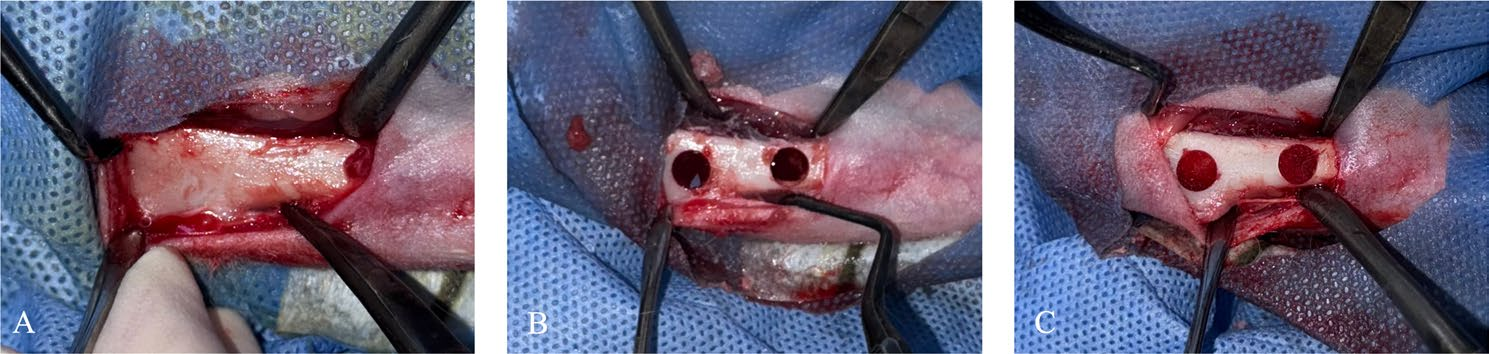
Surgical procedure and defect creation. **A** Exposure of the tibial surface following soft tissue dissection. **B** Creation of two standardized monocortical defects (6 mm diameter, 8 mm depth) with a 10 mm inter-defect distance. **C** Application of treatments: one defect filled with GS (control) and the adjacent defect filled with DXP-impregnated GS

A 2 cm longitudinal incision was made on the anteromedial aspect of the proximal diaphysis of the right tibia, followed by dissection through the skin, fascia, and periosteum to expose the bone surface (Fig. 2A). Two standardized monocortical cylindrical defects (6 mm in diameter, 8 mm depth) were created using a trephine bur (Medicon CMS, Tuttlingen, Germany) under continuous sterile saline irrigation. The second defect was positioned distally with a 10 mm interdefect distance (Fig. 2B).

In each animal, one defect was randomly assigned to the control group and filled with GS, while the adjacent defect was filled with DXP-impregnated GS (Fig. 2C). Soft tissues were closed in layers using 4.0 absorbable polyglactin 910 sutures (Vicryl; Ethicon) for fascia and subcutaneous tissue, and 3.0 Vicryl sutures (Vicryl Plus Antibacterial; Ethicon) [14, 15] for skin closure.

Postoperative care included intramuscular enrofloxacin (2.5 mg/kg) as antibiotic prophylaxis and meloxicam (1 mg/kg) once daily for five days for analgesia. No restriction on food intake or movement was applied, and all animals resumed normal ambulation within 2–3 days without complications.

After a 30-day healing period, animals were euthanized using a high-dose anesthetic consisting of intramuscular ketamine (70 mg/kg) and xylazine (30 mg/kg), in accordance with institutional ethical guidelines. Following euthanasia, tibial specimens were harvested and fixed in 10% neutral buffered formalin. Samples were subsequently decalcified to allow histological processing and were prepared for hematoxylin and eosin (H&E), Picrosirius Red, and immunohistochemical analyses.

### Histomorphological method

All samples were fixed in 10% neutral-buffered formalin for 48 h at room temperature. Histological evaluation was performed by a blinded pathologist to minimize observer bias. After fixation, specimens were decalcified in 10% EDTA solution for two weeks, processed routinely, and embedded in paraffin wax. From each defect, three longitudinal sections were obtained from the central region.

Serial 5 μm-thick sections were stained with hematoxylin and eosin (H&E) for general histomorphological assessment. Histomorphometric analysis included quantitative evaluation of new bone formation (mm²), total healing area (mm²), and defect closure rate (%). Osteoblasts and osteoclasts were counted within a standardized 1.23 mm² field at 400× magnification [16, 17], with measurements performed in five predefined regions per section to ensure sampling consistency. Collagen deposition was evaluated using Picrosirius Red staining (ab150681, Abcam, UK). The intensity of collagen staining was assessed semi-quantitatively using a four-point scale (0 = negative, 1 = mild, 2 = moderate, and 3 = marked collagen deposition).

### Immunohistochemical method

Immunohistochemical staining for vascular endothelial growth factor (VEGF) was performed using a primary antibody against VEGF (#AF5131, Affinity Bioscience, Canada) at 1:100 dilution. After deparaffinization and rehydration, antigen retrieval was carried out using citrate buffer (pH 6.0). Sections were then incubated with the primary antibody at room temperature for 60 min.

Detection was performed using a rabbit-specific horseradish peroxidase (HRP)/DAB detection system (ABC kit, ab64261), followed by incubation with biotinylated secondary antibody and streptavidin–HRP complex according to the manufacturer’s instructions. Between each incubation step, slides were washed twice with phosphate-buffered saline (PBS). Diaminobenzidine (DAB) was used as the chromogen for visualization.

Negative control sections were processed identically, except that the primary antibody was omitted. All immunohistochemical evaluations were performed under blinded conditions to minimize observer bias.

VEGF immunoreactivity was assessed using a semi-quantitative scoring system based on staining intensity: (0 = negative, 1 = mild staining, 2 = moderate staining, and 3 = strong staining). Image acquisition and analysis were performed using an Olympus CX41 light microscope (Olympus Corporation, Tokyo, Japan). Quantitative assessments were conducted using ImageJ software (version 1.48, National Institutes of Health, Bethesda, MD, USA) and CellSens Life Science Imaging Software (Olympus Corporation) following standardized calibration procedures. Following DAB visualization, sections were dehydrated through graded ethanol, cleared in xylene, mounted with a xylene-based mounting medium, and coverslipped for light microscopic evaluation.

### Statistical analyses

Data are expressed as the mean ± standard deviation (SD). Normality of distribution was assessed using the Kolmogorov-Smirnov test, while homogeneity of variances was evaluated with Levene’s test. Given the intra-animal paired design of the study, differences between the control and DXP-treated defects were analyzed using a paired Student’s t-test. Statistical analyses were performed using SPSS software (version 25.0; IBM Corp., Armonk, NY, USA). A p-value < 0.05 was considered statistically significant.

## Results

All samples were successfully processed and analyzed, with no exclusions or losses due to technical issues during decalcification, embedding, or staining procedures.

Histomorphometric analysis demonstrated significantly enhanced bone healing in the DXP-treated defects compared with controls. The total healing area was significantly greater in the DXP group (9.49 ± 0.27 mm²) than in the control group (5.60 ± 0.28 mm²; *P* < 0.001). Similarly, the defect closure rate was markedly higher in the DXP group (59.31 ± 2.22%) compared with controls (48.62 ± 0.84%; *P* < 0.001).

New bone formation was significantly increased following DXP treatment (6.36 ± 0.09 mm²), relative to the control group (4.41 ± 0.23 mm²; *P* < 0.001). Osteoblast counts were also significantly higher in the DXP group (17.00 ± 1.41) than in controls (13.00 ± 1.60; *P* < 0.001), whereas no statistically significant difference was observed in osteoclast numbers between the groups (*P* > 0.05).

Collagen deposition, assessed by Picrosirius Red staining, was significantly greater in the DXP group (2.62 ± 0.51) compared with the control group (1.50 ± 0.53; *P* < 0.001) indicating enhanced matrix organization.

**Fig. 3:**
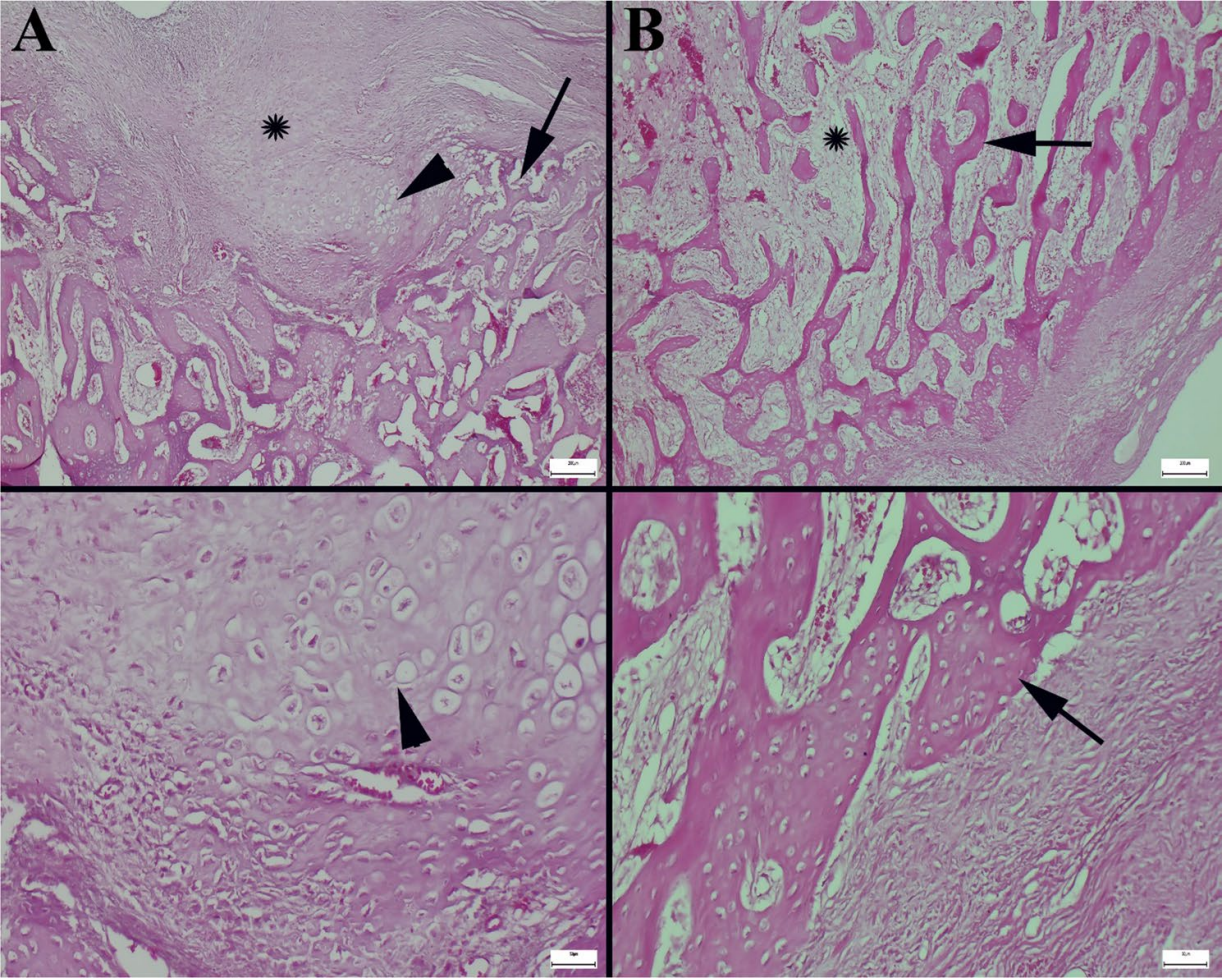
Representative histological sections of defect areas at low (upper row) and high magnification (lower row). **A** Control group showing prominent fibrous tissue (asterix), cartilage formation (arrowheads), and limited new bone formation (arrows). **B** DXP-treated group demonstrating reduced fibrous tissue and increased newly formed bone (arrows). H&E staining; scale bars = 200 μm (upper panels) and 50 μm (lower panels)

**Fig. 4:**
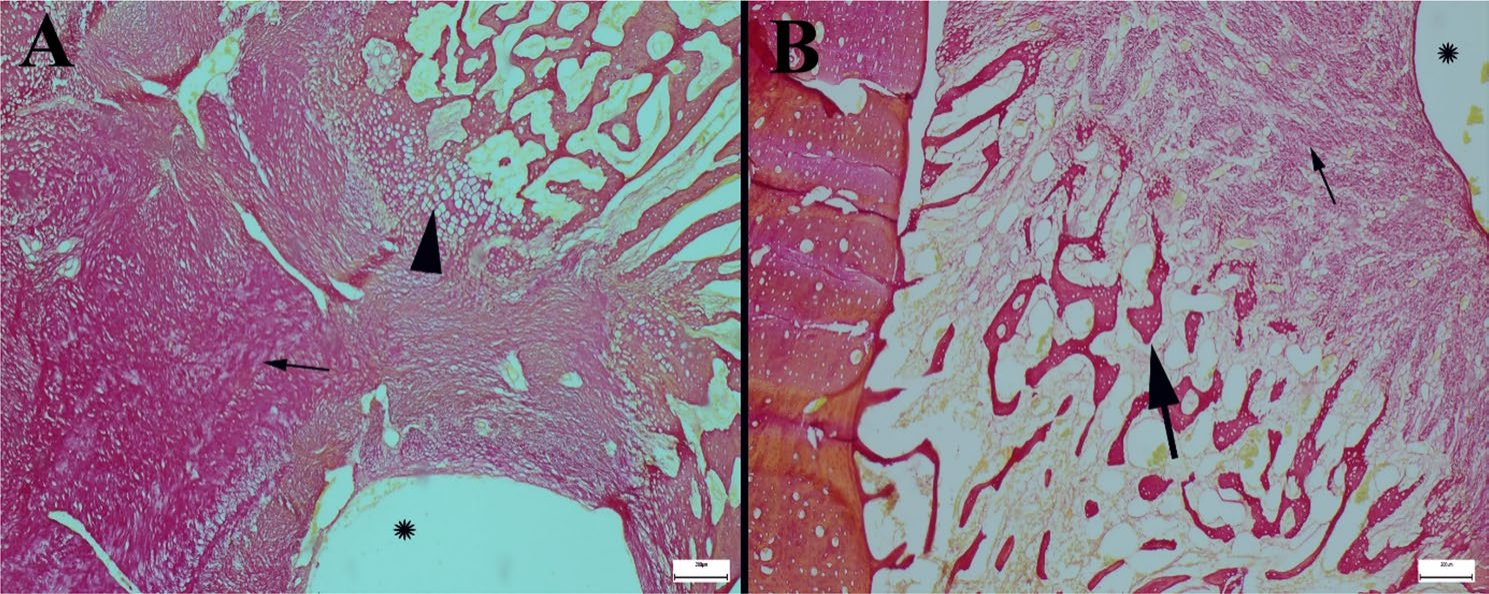
Collagen deposition in defect areas. **A** Control group showing moderate collagen deposition (thin arrow) and areas of cartilage formation (arrow head) around the defect area (asterix). **B** DXP-treated group demonstrating increased and more organized collagen deposition (thin arrow), associated with new bone formation (thick arrow) around the defect area (asterix). Picrosirius Red staining; scale bars = 200 μm

**Fig. 5:**
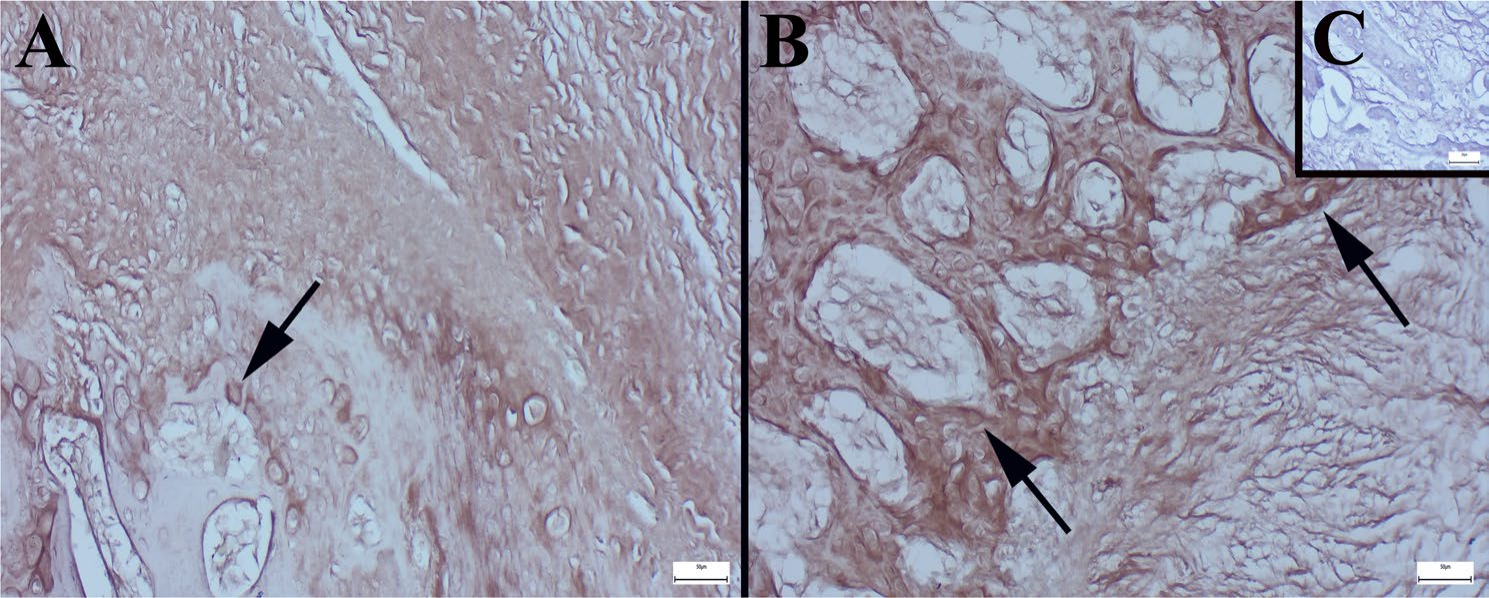
Representative images of VEGF immunostaining in the defect areas. **A** Control group showing mild to moderate VEGF immunoreactivity. **B** DXP-treated group demonstrating increased VEGF expression. **C** Inset showing the negative control processed without the primary VEGF antibody. Streptavidine biotine HRP method with DAB cromogen; scale bar = 50 μm

Immunohistochemical analysis revealed significantly increased VEGF expression in the DXP group (2.25 ± 0.46) compared with controls (1.50 ± 0.25; *P* < 0.001), suggesting an enhanced angiogenic response in DXP-treated defects (Figs. 3, 4 and 5; Table 1).

**Table 1:** Histomorphometric and immunohistochemical comparison between control and dexpanthenol-treated defects

Parameter	Control (*n* = 8)	DXP (*n* = 8)	*P* value^*^
Total healing area (mm^2^)	5.60 ± 0.28	9.49 ± 0.27	**< 0.001**
Defect closure rate (%)	48.62 ± 0.84	59.31 ± 2.22	**< 0.001**
New bone formation area (mm^2^)	4.41 ± 0.23	6.36 ± 0.09	**< 0.001**
Osteoclasts count	2.50 ± 0.92	2.12 ± 0.83	> 0.05
Osteoblasts count	13.00 ± 1.60	17.00 ± 1.41	**< 0.001**
Collagen deposition (score)	1.50 ± 0.53	2.62 ± 0.51	**< 0.001**
VEGF expression (score)	1.50 ± 0.25	2.25 ± 0.46	**< 0.001**

Values are presented as the mean ± standard deviation (SD). All comparisons were performed using a paired Student’s t-test, reflecting the intra-animal paired study design. Collagen deposition and VEGF expression were evaluated using a semi-quantitative scoring system (0 = negative, 1 = mild, 2 = moderate, 3 = strong). Statistically significant differences were considered at *P* < 0.05. Bold values indicate statistically significant differences (*P* < 0.05)

*Abbreviations*: *DXP* dexpanthenol, *VEGF* vascular endothelial growth factor

Histologically, DXP-treated defects demonstrated more advanced bone regeneration characterized by extensive new bone formation and reduced immature connective tissue compared with controls.

## Discussion

Bone tissue possesses a well-orchestrated regenerative capacity governed by the balance between bone formation and osteoclastic resorption. However, this intrinsic healing potential may be insufficient in cases of trauma, infection, or systemic impairment, necessitating the use of bioactive agents to enhance regeneration [18]. In the present study, locally delivered DXP significantly improved histomorphometric bone healing parameters and increased VEGF expression in a rabbit tibial defect model.

DXP is a well-tolerated compound widely used in soft tissue repair, where it exerts antioxidant and anti-inflammatory effects and supports cellular metabolism [9, 10]. While its beneficial effects on cutaneous and mucosal wound healing are well established [6], its role in bone regeneration has remained largely unexplored. Our findings demonstrate that DXP significantly enhances new bone formation, defect closure, and collagen deposition, suggesting a broader regenerative potential beyond soft tissues.

GS was used as a carrier due to its biocompatibility, biodegradability, and porous structure, which supports cell infiltration and matrix deposition [19]. Although GS alone has been reported to promote early connective tissue and cartilage formation [20], our control group findings were consistent with this pattern, showing predominance of fibrous tissue and limited bone formation. In contrast, DXP-treated defects demonstrated a clear shift toward organized bone regeneration, indicating that DXP contributes to the transition from early reparative tissue to mature bone formation.

The significantly increased collagen deposition observed in the DXP group aligns with previous reports demonstrating its stimulatory effects on fibroblast activity and extracellular matrix synthesis in soft tissues [7]. Since collagen-rich matrix formation is a prerequisite for mineralized bone deposition, these findings suggest that DXP may indirectly facilitate osteogenesis by enhancing the early extracellular scaffold required for bone maturation.

Although the precise molecular mechanisms remain unclear, DXP has been reported in other biological systems to modulate inflammatory and profibrotic pathways, including TNF-α, TGF-β signaling [21–24]. In the present study, osteoblast counts were significantly increased in the DXP group, while osteoclast numbers remained unchanged. This suggests that DXP may favor bone formation without altering resorptive activity, thereby supporting a net anabolic environment. Consistent with these observations, experimental agents such as Withaferin-A and salubrinal have similarly been reported to enhance osteogenesis and modulate osteoclast activity through distinct molecular pathways [24, 25], further supporting the notion that pharmacological modulation of bone cell activity can yield favorable regenerative outcomes. However, the absence of direct molecular analyses limits mechanistic interpretation.

Angiogenesis plays a critical role in bone regeneration, and VEGF is a key mediator of this process. In this study, VEGF expression was significantly upregulated in DXP-treated defects, indicating enhanced angiogenic activity. This finding is consistent with evidence that antioxidant-related signaling can upregulate VEGF expression and promote vascularization [26–28]. Given the coupling between angiogenesis and osteogenesis, improved vascular response may represent a central mechanism underlying the enhanced bone healing observed in this study.

From a translational perspective, local delivery of DXP may represent a promising adjunct in oral and maxillofacial regenerative procedures, where rapid vascularization and matrix formation are essential for graft integration [29]. Its incorporation into scaffold-based systems may further enhance early healing responses. However, the release kinetics of DXP from gelatin sponge were not evaluated, and whether its effect is driven by burst or sustained release remains unknown, limiting direct clinical extrapolation.

Several limitations should be acknowledged. First, the study was limited to a single time point, preventing evaluation of temporal healing dynamics. Second, only VEGF was assessed at the molecular level, without analysis of additional osteogenic or inflammatory markers. Third, micro-computed tomography was not performed, limiting three-dimensional assessment of bone architecture [30]. Fourth, the paired defect design, while reducing inter-animal variability, may have introduced potential local biological interaction between defects, as suggested by the reviewer, and an alternative design with one defect per tibia could potentially eliminate any influence related to the close proximity of defects. Fifth, collagen subtype differentiation (e.g., type I vs. type III collagen) was not performed, which limits the ability to fully characterize the maturation and quality of the newly formed extracellular matrix. Sixth, the use of a tibial defect model differs substantially from oral and maxillofacial bone in both anatomical and biological aspects, thereby limiting the direct clinical relevance and extrapolation of the findings. Finally, the absence of a DXP-only group precludes differentiation between the effects of DXP and its gelatin sponge carrier, making it difficult to determine whether the observed outcomes are additive or synergistic.

## Conclusions

Within the limitations of this experimental rabbit tibial defect model, local administration of DXP was associated with enhanced bone regeneration, as evidenced by increased new bone formation, collagen deposition, osteoblast activity, and VEGF expression compared with the control defects. Collectively, these findings suggest that DXP may positively modulate early bone healing by promoting osteogenic and angiogenic responses. However, the lack of three-dimensional imaging, release kinetics evaluation, and molecular pathway investigations limits a comprehensive understanding of its underlying mechanisms and translational relevance. Future studies using clinically relevant alveolar or mandibular defect models, combined with advanced imaging and molecular techniques, are required to elucidate the therapeutic potential of DXP in oral and maxillofacial bone regeneration. 

## Data Availability

The datasets used and analysed during the current study available from the corresponding author on reasonable request.

## Abbreviations

DXP: Dexpanthenol
GS: Gelatin sponge
HE: Hematoxylin and eosin
VEGF: The vascular endothelial growth factor
DAB: Diaminobenzidine
